# **Purpose, Subject, and Consumer** Comment on "Perceived Burden Due to Registrations for Quality Monitoring and Improvement in Hospitals: A Mixed Methods Study"

**DOI:** 10.34172/ijhpm.2022.6495

**Published:** 2022-02-09

**Authors:** Sylvia J. Hysong, Patrick O’Mahen, Jochen Profit, Laura A. Petersen

**Affiliations:** ^1^VA HSR&D Center for Innovations in Quality, Effectiveness and Safety, Michael E. DeBakey VA Medical Center, Houston, TX, USA.; ^2^Department of Medicine, Health Services Research Section, Baylor College of Medicine, Houston, TX, USA.; ^3^Epidemiology and Health Outcomes Research Unit, Division of Neonatology, Department of Pediatrics, Stanford University School of Medicine and Lucile Packard Children’s Hospital, Palo Alto, CA, USA.; ^4^California Perinatal Quality Care Collaborative, Palo Alto, CA, USA.

**Keywords:** Clinical Performance Measurement, Quality Monitoring Burden, Purpose-Subject-Consumer Framework, Performance Measure De-implementation, Clinicians

## Abstract

Zegers and colleagues’ study codifies the perceived burden of quality monitoring and improvement stemming from the work by clinicians of registering (documenting) quality information in the medical record. We agree with Zegers and colleagues’ recommendation that a smaller, more effective and curated set of measures is needed to reduce burden, confusion, and expense. We further note that focusing on validity of clinical evidence behind individual measures is critical, but insufficient. We therefore extend Zegers and colleagues’ work through a pragmatic, tripartite heuristic. To assess the value of and curate a targeted set of performance measures, we propose concentrating on the relationships among three factors: (1) The *purpose* of the performance measure, (2) the *subject* being evaluated, and (3) the *consumer* using information for decision-making. Our proposed tripartite framework lays the groundwork for executing the evidence-based recommendations proposed by Zegers et al, and provides a path forward for more effective healthcare performance-measurement systems.

 Performance measurement and quality monitoring are ubiquitous in healthcare. In the last two decades, measuring performance grew from something other industries did to becoming the nexus of many of the decisions we make in healthcare, from selecting the clinicians and facilities with the best quality of care for a given condition, to maintaining Joint Commission accreditation, to optimizing patient panels for insurance purposes. What began as a good idea, however, has proliferated into a growing problem: an overabundance of measures. In the United States alone, the National Quality Forum catalogs 1086 clinician-focused performance measures.^1^ In their empirical study, Zegers and colleagues^2^ contribute to the quality measurement literature by codifying and quantifying what healthcare professionals experience every day: the palpable burden of quality performance measurement. as described in their findings, clinicians perceived the number of measures and the time required to document (register) them in the medical record as excessive, and quality improvemen registration was perceived as taking time away from the patient experience. Most importantly, only 36% of measures required were perceived to aid in quality improvement. Faced with what feels like an excessive burden, clinicians and patients often suffer unintended consequences from quality measurement: clinicians struggle to prioritize among competing quality-improvement initiatives, and patients become confused when making informed health decisions.^3-5^ We therefore agree with Zegers that such a beehive of quality measures burdens both clinicians and patients and agree that a smaller, more focused, efficient, and effective set of measures is clearly needed to preserve clinicians’ intrinsic motivation and autonomy, and also reduce burden, confusion, and expense.^4,6^ The problem is deciding what to include in this smaller set.

 Previous frameworks and approaches to quality measure development and evaluation, such as those proposed by Evans and colleagues,^7^ Stelfox and Straus,^8,9^ and others, have approached quality measure development and selection from the perspective of perfecting a given measure by vetting against stricter or more nuanced quality criteria,^3,10-13^ or of eliminating clinically unnecessary measures (such as those based on clinical practices identified in the Choosing Wisely campaign).^14^ Others attempt to decrease burden through prioritizing existing measures^15^; none of these approaches, however – all recommended by Zegers and colleagues – convey to clinicians the value of these quality measures to their daily practices. Consequently, they and other stakeholders still puzzle through how best to use existing measures to achieve clinical and professional objectives, thus increasing perceived burden. As a practical service to clinicians and stakeholders, we present a straightforward heuristic for prioritizing relevant (and eschewing low-value) measures, thus extending Zegers and colleagues’ work.

## A Framework for Strategically Selecting Performance Measures

 Pronovost^16^ posited that the reason there is so much variability in what constitutes high- quality care is that there is no clarity or consensus on the purpose of healthcare. We similarly contend one the reason for the current measure proliferation (and accompanying burden) is a lack of clarity on their purpose and intended value. For the purposes of this discussion, we define performance in terms of constructs advocated by Kane^17^ and echoed by the National Academies^18^: quality, quantity, timeliness, cost effectiveness, need for supervision and interpersonal impact.

 To create or identify a targeted set of performance measures, we propose concentrating on the relationships among three factors: (1) for what *purpose* is performance information being used?, (2) who is the *subject* being evaluated?, and (3) who is the *consumer *using information for decision-making? If clear, specific answers to these questions cannot be crafted about an existing or proposed measure, then the measure should not be implemented. Assuming clear, specific answers are possible, said answers can strategically drive *selection* of appropriate measures by clarifying the types of questions answerable with measures of a specific purpose/subject/consumer combination (see Supplementary file 1 for examples), which can help reduce information overload and maximize value. More detailed, follow-up questions can *then* be asked (such as the logistics of operationalization and data capture) – and indeed, are suggested by others, eg, Stelfox and Straus^8,9^; Pritchard et al^19^ – to further refine the operationalization and filtering process if desired. Below we expand on each factor, followed by an example application of the framework. Details on the development of this framework can be found in Supplementary file 1.

## Purpose

 Performance information serves numerous purposes. Aguinis^20^ posits six: *strategic, administrative*, *communication, developmental, organizational maintenance, and documentation*; the Table below presents brief definitions of these. The first three purposes are consistent with existing healthcare-specific taxonomies such as those employed by the Measurement Applications Partnership.* Organizational maintenance *and* documentation *could be considered special cases of strategic and administrative purposes, respectively; thus, we expand below on only the first four.

**Table:** Purposes of Performance Information and Their Definitions

**Purpose**	**Description**
Strategic	Linking organizational goals with individual goals, to reinforce behaviors consistent with organizational goals.
Administrative	Making between-provider or between-organization comparisons to make administrative decisions such as selection, termination, merit increases.
Communication	Informs the evaluee how well he or she is doing, areas for improvement, and communicates expectations.
Developmental	Includes feedback, intended for coaching individuals on and helping them improve performance on an ongoing basis.
Organizational Maintenance	Gives targets information useful for workforce planning or future strategy.
Documentation	Yields data that can be used to assess the predictive accuracy of newly proposed selection instruments, as well as important administrative decisions (particularly useful for litigation).

Note: Adapted from Aguinis.^20^

 When developing measures for *strategic* purposes, the objective is to clarify the connections between organizational and individual goals to ensure that clinician behavior aligns with organizational objectives. For example, measures tracking hospital patients with central line-associated bloodstream infection assess clinical outcomes *strategically* aligned with the organizational goalof improving inpatient safety and avoiding financial penalties.

 Performance measures serve an *administrative* purpose when used to make inter-clinician or inter-organizational comparisons of performance that inform organizational decisions like personnel selection, termination, promotion, or compensation. For example, with the Centers for Medicaid and Medicare Services implementing more pay-for-performance incentives like those contained within the Medicare Access and Children’s Health Insurance Program Reauthorization Act, performance measures increasingly serve as administrative benchmarks for determining compensation.

*Communication *purposes evolve from a focus on individual improvement, specifically, conveying performance expectations to evaluees while suggesting areas for improvement. Many clinician dashboards, for example, serve this purpose when they display individual clinician scores on preventive care measures such as immunizations or screening rates. When a performance measure is used for coaching and performance improvement, with no links to administrative purposes (for example, tracking patient-centered communication behaviors in clinicians to help them improve rapport with patients), it is considered a developmental purpose.

 Any given measure could conceivably serve multiple purposes; according to Zegers et al, such a measure would be highly desirable for its efficiency and economy of data collection. For example, tracking immunization rates could serve both the strategic purpose of aligning individual clinicians’ behaviors with a facility’s public health mission, the communication purpose of informing clinicians about their current performance and expected targets, and the administrative purpose of awarding merit increases to the highest performers. The two other factors, subject and consumer, can provide further guidance on the suitability of a measure for a given purpose.

## Subject

 Performance information in healthcare is largely collected from three basic subject types: clinicians, facilities, and payers, and clarity on the subject of the measure in question is critical. *Clinicians* often represent the basic unit of analysis, delivering care and serving as fundamental interfaces between healthcare and patients; clinician performance scores aim to assess clinical effectiveness. However, in team-based clinical settings, using individual clinicians as the unit of analysis may be inappropriate.* Facilities’ performance is* often reflected as aggregates of clinician or work-unit performance scores. However, facilities vary by characteristics such as configuration (eg, general vs. specialty hospital), infrastructure (on-site vs outsourced laboratory services), teaching mission (healthcare trainees onsite) and organizational culture (hierarchical vs. decentralized teams). These characteristics are unique to facilities, and vary in facility-specific ways to influence facilities’ adeptness at delivering high-quality, cost-effective, patient-centered care, and yield useful facility-level performance information; for example, evaluating amount and type of communication, shared values, and cooperation among clinicians. Finally, performance information covers *payers*. Patients desire payers that are cost effective, pay claims in a timely manner, and facilitate access to care. Similarly, clinicians and facilities appreciate payers that reimburse adequately, with minimal administrative burden.

## Consumer

 Consumers are users requiring quality/performance information for decision-making. Importantly, any subject of a performance measure (clinicians, facilities, payers) could themselves be a consumer. Clinicians, for example, consume performance information to make decisions about patients (eg, treatment adherence), themselves (clinical skills), colleagues (referrals), their facility (staff responsiveness), or payers (insurer claim resolution speed). Consumers may highly value the satisfaction ratings of other consumers (eg, star ratings). Other entities, such as accrediting organizations or regulatory agencies, also have specific information needs. Each consumer type needs different information, such as timeliness, cost-effectiveness, and interpersonal impact, requiring a narrow set of situationally appropriate measures.

 In summary, we propose that ascertaining a proposed performance measure’s purpose, and target subject and consumer serves as an initial needs assessment to identify a measure’s value, which serves as a powerful criterion by which to discard redundant or irrelevant measures.

## Applying the Framework

 To illustrate how the framework can address Zegers and colleagues’ recommendations and help reduce clinician burden, consider a fictitious example. Dr. Smith, the chief of gastroenterology at a teaching hospital, faces increasing pressure to deliver more clinical value (higher care quality for a given cost). That pressure may stem from government regulatory audits, or from private insurers (in countries that have them). She relies on performance measures to help guide her decision-making. But which measures should she use? It goes without saying that selected measures should be valid and accurately reflect the process or outcome being assessed^8,9,18^; however, although many advances have been made on this front, measure validity is a necessary but not sufficient condition for arriving at an *efficient* set of measures that accomplish their goals in a parsimonious manner. Dr. Smith’s top priorities are to align her clinicians’ outcomes with the goal of increased value but also to help them improve their skills as patient-centered clinicians. Dr. Smith’s *purpose* for performance measurement is both strategic and developmental. Because her interest is in changing clinicians’ behavior, the subject in question is the clinician; because there are both strategic and developmental purposes at play, both the individual clinician (for developmental purposes) and the clinic leadership (for strategic purposes) are consumers of performance measure information. Thus, Dr. Smith needs a concise set of clinician-level measures that can help forward her strategic goals; her clinicians need an equally concise set of clinician-level measures that can help achieve their developmental goals toward patient centeredness.

 Rather than overwhelm her clinic with a broad spectrum of measures, she chooses to focus her team on the clinical areas where gastroenterology can provide most value: colon cancer screening and follow-up colonoscopy screening tests, which her department provides onsite. By improving the consistency of these services, diagnoses and plans of care can be made more quickly and effectively for patients; and she can reduce the potential for missed diagnoses without adding cost compared to offsite referrals.

 Measures addressing Dr. Smith’s strategic purpose of increasing value could include: (1) patient ratings of value or satisfaction; (2) individual clinicians’ missed opportunities for care coordination (missed coordination means duplication of work and, thus, lower output for the increased cost); (3) key measures on the specific clinical areas of focus, such as colon cancer and follow-up colonoscopy screening rates; and (4) a more direct ratio of quality over cost, such as the percent of routine comprehensive physicals with lab testing performed on otherwise healthy adults. The set of measures clinicians should receive, given their purpose, however, is slightly different. They could receive measures 2-4 to help alert them to areas of value where they could contribute and improve, thereby serving Dr. Smith’s strategic goals. The care coordination measure in particular could help the developmental goal of improving patient-centered care, by alerting clinicians to a specific patient-centered concern that may warrant attention. However, A simple patient satisfaction rating (a very common measure) would not provide sufficient information to the clinician to facilitate behavior change and would not be worth presenting to that consumer by itself, without information on what to change. Similarly, a measure specifically captured for clinicians’ development could include a measure such as the percent of encounters in which the clinician exhibited appropriate communication behaviors, (as measured perhaps through a mystery shopper approach). This would be highly useful information for individual clinicians to facilitate behavior change but would provide little useful information to Dr. Smith at the strategic level.

 As shown in our example, applying our proposed framework acts as a form of high-level needs assessment (a step advocated but often overlooked in measure development and implementation).^8,9,18^ Defining a clear, specific purpose helps identify key outcomes that need to be measured, whittling down potentially relevant measurements. Clearly recognizing who is the subject of measurement helps identify the correct unit of aggregation for the measures, which can eliminate additional irrelevant measures. Finally, considering for what purpose consumers will be using the measures and what decisions the measures might inform will help determine the most appropriate type of measure to select. Combined, the three factors identify the need the measure(s) addresses and the value added by implementing it, which acts as a powerful mechanism to filter out redundant or irrelevant measures, thus reducing the overall number of performance measures.

 Moreover, aside from any reduction in the raw number of measures, using the framework generates a secondary benefit: it encourages administrators to integrate clinicians and other front-line medical staff into the decision-making process for selecting measures. This bottom-up process can provide transparency and clarity, which can promote clinicians’ recognition of the measures’ value^21,22^ and thus reduce perceptions of burden such as those observed by Zegers and colleagues regardless of the actual number measures. Although this example deals with a hospital specialty department, the heuristic can be adapted and used by anyone in the healthcare system – a hospital system, a public health official or even a prospective patient.

## Conclusion

 Our proposed framework lays the groundwork for executing the evidence-based recommendations proposed by Zegers et al, and provides a path forward for more effective healthcare performance-measurement systems. Focusing on validity of clinical evidence behind individual measures is critical, but insufficient. Whether reconsidering extant measures or developing new ones, our proposed framework of subject, consumer, and purpose can help align measures with their intended decision-making and behavior-change goals, while empowering both institutions that evaluate and those needing evaluations. The result can be a more efficient, effective health system that better serves clinicians, payers and patients and a positive step toward alleviating the burden Zegers et al have so helpfully quantified.

## Ethical issues

 Not applicable.

## Competing interests

 Authors declare that they have no competing interests.

## Authors’ contributions

 SJH conceptualized the framework, conducted the data analysis in and prepared Supplementary file 1, assumed principal writing responsibility for the manuscript (initial draft and revisions). PO contributed material scientific content, and assumed principal editing responsibility for the manuscript (scientific content, clarity of ideas, and style). JP provided material scientific content and style edits to all versions of the manuscript. LAP provided material edits to early drafts of the framework, and provided material scientific content and style edits to all versions of the manuscript.

## Disclaimer

 The views expressed in this article are those of the authors and do not necessarily represent those of the Veterans Health Administration or the US Government.

## Funding

 This material is based upon work supported in part by the Department of Veterans Affairs, Veterans Health Administration (VHA), Office of Health Services Research and Development, and the Center for Innovations in Quality, Effectiveness and Safety (grant number CIN-13-413), as well VHA grants CDA 07-0181, CRE 12-035 and IIR 15-438.

## Authors’ affiliations

^1^VA HSR&D Center for Innovations in Quality, Effectiveness and Safety, Michael E. DeBakey VA Medical Center, Houston, TX, USA. ^2^Department of Medicine, Health Services Research Section, Baylor College of Medicine, Houston, TX, USA. ^3^Epidemiology and Health Outcomes Research Unit, Division of Neonatology, Department of Pediatrics, Stanford University School of Medicine and Lucile Packard Children’s Hospital, Palo Alto, CA, USA. ^4^California Perinatal Quality Care Collaborative, Palo Alto, CA, USA.

## Supplementary files


Supplementary file 1. Developing the Purpose-Subject-Consumer Framework.
