# Supplementary material for: Purpose, Subject, and Consumer Comment on "Perceived Burden Due to Registrations for Quality Monitoring and Improvement in Hospitals: A Mixed Methods Study"
Source: Int J Health Policy Manag. 2022 Feb 9;11(4):539–43. doi: 10.34172/ijhpm.2022.6495 (PMC9309954; doi:10.34172/ijhpm.2022.6495)
Supplement: Supplementary file 1 — Developing the Purpose-Subject-Consumer Framework. [file ijhpm-11-539-s001.pdf]

Article title: Purpose, Subject, and Consumer; Comment on "Perceived Burden Due to Registrations for Quality Monitoring and Improvement in Hospitals: A Mixed Methods Study"

Journal name: International Journal of Health Policy and Management (IJHPM)

Authors' information: Sylvia J. Hysong[1,2]*, Patrick O'Mahen[1,2], Jochen Profit[3,4], Laura A. Petersen[1,2]

[1]VA HSR&D Center for Innovations in Quality, Effectiveness and Safety, Michael E. DeBakey VA Medical Center, Houston, TX, USA.

[2]Department of Medicine, Health Services Research Section, Baylor College of Medicine, Houston, TX, USA.

[3]Epidemiology and Health Outcomes Research Unit, Division of Neonatology, Department of Pediatrics, Stanford University School of Medicine and Lucile Packard Children's Hospital, Palo Alto, CA, USA.

[4]California Perinatal Quality Care Collaborative, Palo Alto, CA, USA.

(*Corresponding author: Sylvia.hysong@va.gov)

Supplementary file 1. Developing the Purpose-Subject-Consumer Framework

The purpose – subject – consumer framework offers a pragmatic needs assessment for prioritizing quality measures. It arose from the need to create a way to organize and focus the overwhelming number of quality measures currently in existence so that they can have their intended effect. It also stemmed from our observation that many performance measure development frameworks in the literature , including some highly respected ones (such as the National Quality Forum), concentrated their efforts on the technical details of the measure, while not necessarily paying attention to a measure's incremental contribution in the performance domain.[1-3] Beginning "with the end in mind", our straightforward approach is consistent with multiple organizational change and quality improvement frameworks, such as the Productivity Measurement and Enhancement System,[4] Design Thinking,[5] Understanding by Design,[6] and classic performance management.[7, 8] To test the viability of this pragmatic framework on existing performance measures we conducted a thought exercise; the authors generated a set of questions that could be answered by a specific purpose-subject-consumer combination. We

followed our thought exercise with a categorization of existing quality measures endorsed by the National Quality Form (NQF), to test how well the 6 purposes proposed by Aguinis could be used to categorize existing healthcare metrics.

Thought Exercise: Questions Answerable Using the Purpose-Subject-Consumer Framework

Though traditionally performance measures have been associated with clinical quality, health care performance can encompass a much broader range of variables, which partially accounts for the existing proliferation of measures. Given these three factors (subject, consumer, and purpose), the specific types of questions a consumer would want answered about a subject can be specified and classified by purpose. A sample of such a classification appears in Table S1. As can be seen from the table, the most common question the consumer is trying to answer is one of selection: Which facility should I go to for my procedure? Which payer should the hospital list with? Which hospital best fits the consumer's needs? Although other question types are possible (e.g., identification: In what areas do I need to improve as a clinician/ hospital / payer?) the observation that selection is the most common type of question answerable through specific purpose/subject/consumer combinations suggests the framework is viable as a means of reducing the number of measures required by a practice, thereby reducing burden and cost.

The table also illustrates that not every purpose is applicable or pertains to every subject-consumer combination. For example, based on the table, there is no performance information a patient would need about a clinician, facility, or payer that serves an organizational maintenance purpose; as their name implies, organizational maintenance measures are purely for organizations to plan future strategy. Therefore, in this way the framework also serves as a way to focus performance measure development efforts.

Measure Categorization: Applying the Purpose-Subject-Consumer Framework to Existing Measures

To illustrate our framework on existing quality measures, we sought to determine the variety of purposes for which existing measures are currently used. We used a sample of measures in the Agency for Healthcare Research and Quality (AHRQ) Quality Measures Clearinghouse endorsed by the NQF, which we attempted to categorize, using our framework.

We queried the AHRQ Quality Measures Clearinghouse in June 2014 to retrieve all available quality measures, restricted only to measures endorsed by the NQF; we used this restriction to ensure retrieval only of well-developed quality measures.5 NQF endorses only measures that follow strict development criteria, such as validity and reliability, impact, feasibility, and usability.14 Data elements available for each measure include agency/division (e.g., AHRQ, Health Resources and Services Administration, National Institutes of Health), topic or condition (e.g., patient safety, diabetes), measure domain (e.g., process vs. outcome), setting (e.g., impatient vs. ambulatory), measure title, definition, and purpose (e.g., pay-for-reporting, quality improvement, benchmarking).

To categorize the measures by purpose, we examined the stated purpose of the NQF-endorsed measure in the database and classified each stated purpose into one of the six purposes proposed by Aguinis,12 based on their definition. We used the Recode procedure in SPSS 22.0 to automatically assign Aguinis purposes to individual measures, based on their stated purpose. Any measures not captured by the automatic recode process were recoded manually. We attempted to manually categorize measures into subject type and consumer type, based on the titles and definitions available in the database.

Applying our selection criteria resulted in 672 measures; Table S2 presents the NQF-endorsed measures by purpose and clinical topic. It was not possible, based on data from the AHRQ clearinghouse, to discern the intended consumer or subject of the measures. As can be seen from the table, cardiovascular (n=101), patient safety (n=91), and communicable diseases (n=71) contain the greatest number of unique measures,† accounting for 39% of the 672 NQF-endorsed measures observed. The most common purpose for measurement by far is administrative — 68% of cardiovascular, 86% of patient safety, 44% of communicable disease, and 70% of all NQF-endorsed measures serve one or more administrative purposes, such as pay for reporting. Notably, only 2% of available NQF-endorsed quality measures served a strategic purpose, as defined by Aguinis (such as benchmarking, national tracking and trending).

Our exploration into existing NQF measures (among the most well-documented and accepted health-care measures available) suggests that considerable gaps exist in available

† Five measures were categorized as both cardiovascular and patient safety; these have been removed from the cardiovascular count of 99 in the text (i.e., total # of measures categorized with the label cardiovascular = 104).

measures to fulfill a several of Aguinis' purposes (e.g,. no quality measures were found for organizational maintenance purposes, and less than 3% of measures were used for developmental purposes -- the main source of dissatisfaction expressed by participants in the Zegers et al. article). Though many of these measures are evidence based in terms of clinical efficacy and focus on highly prevalent clinical areas representing a considerable portion of the national disease burden, the number of measures available within a given purpose and/or condition suggests an opportunistic, rather than a strategic, approach to measure development. These findings, combined with the results of our thought exercise, led us to conclude that the purpose-subject-consumer framework could be used as proposed: as a form of initial needs assessment to establish the incremental value or contribution of a proposed measure or measures, before further exploring the clinical and logistical details of their development.

Table S1. Possible Purposes of Performance Information, by Subject and Consumer

Consumer of Performance Information		Purpose	Patient	Clinician	Facility	Payer
			Subject of Performance Information			
	**Patient**	*Strategic*	• Are my behaviors in line with my health goals?	• Are my clinician's health goals for me aligned with my health goals?	• Does the facility provide a good health-care experience?	• **Selection**: which payer do I choose? Are my payer's cost and coverage goals aligned with mine?
	**Patient**	*Administrative*		• **Selection**: Which doctor do I choose?<br>• Do I have easy access to my clinician	• **Selection**: Which hospital provides the highest quality of services for my condition? satisfaction)	• Are my claims paid with minimal hassle?
	**Patient**	*Developmental*	• **Identification:** In what areas can I improve?	• Does this clinician coordinate my care well?		
	**Patient**	*Organizational Maintenance*				
	**Patient**	*Communication*				
	**Patient**	*Documentation*	• Are my medical records available to me?	• Can I access my own medical record (EHR)?		• Can I easily access my claims?
	**Clinician**	*Strategic*	• Is my patient panel in line with evidence-based care goals? (panel management)		• What is the hospital's vision? Strategy?	• Does the payer have sufficient market penetration in my area to attract new patients?

	Administrative			• **Selection**: At what hospital do I want to have privileges?	• How restrictive is the payer's formulary?<br>• Are my claims paid on time?
	Developmental		• **Identification:** What clinical skills do I need to improve upon?<br>• How will I be evaluated?	• What are the facility performance-management benchmarks?	
	Organizational Maintenance				
	Communication		• What areas does my organization value?		
	Documentation		• Legal defense		
**Facility**	*Strategic*	• What services can I provide that will best fit my patient population's needs?	• How do I best align our clinicians' behavior with our vision and strategy	• How do I compare against other facilities like me?	• Does the payer have sufficient market penetration in my area to attract new patients?
	Administrative		• Selection, promotion, raises, incentives: Who are my best employees and how do I keep them?	• Are we meeting Joint Commission Accreditation standards?	• What other hospitals (competitors) list with a given payer?<br>• How restrictive is the payer's formulary?<br>• Are my claims paid on time?

	Developmental	• How satisfied are our patients with the quality of services and care we provide?	• In what areas do my clinicians need to improve?	• In what areas does the hospital need to improve (e.g., falls, safety, infections)?	
	Organizational Maintenance		• What skill mix does my hospital need?	• History and trends for forecasting	
	Communication		• What principles do we value?		
	Documentation		• Legal defense	• Comply with federal reporting requirements	
**Payer**	*Strategic*	• What type of patients do I need to enroll to optimize my pool of insured patients?	• **Advertising** – e.g., do the payer's in-network clinicians meet a specified threshold of quality they can advertise?	• Advertising<br>• Pay-for-quality: Does the facility meet criteria for quality bonuses?	• In what markets do I need better penetration?
	Administrative		• **Selection**: which clinicians will I contract with?	• P4P, selection, regulation (with which hospitals will I contract?)<br>• Never events-CMS	
	Developmental	• How can I help enrolled patients to engage in more preventive care?			• How can process claims more efficiently?
	Organizational Maintenance				• Legal defense
	Communication				• Financial management
	Documentation				• Legal defense

		Other				• Financial management

Blank cells indicate purposes that are not applicable to a given subject – consumer combination.

EHR = electronic health record;

Table S2. National Quality Foundation-endorsed Measures, Categorized by Measure Purpose and Clinical Topic

Topic or Condition	Purpose							
	Administrative	Developmental	Documentation	Org. Maintenance	Conflicting	Unknown	Total	% of Grand Total
Blood Products/Transfusion	1	0	1	0	0	2	4	0.60
Cancer	27	4	10	0	1	1	43	6.40
Cardiovascular	71	5	21	0	1	6	104	15.48
Cerebrovascular	21	0	2	0	0	0	23	3.42
Chronic & Elder Care	18	0	0	0	2	3	23	3.42
Communicable Diseases	32	1	6	0	33	0	72	10.71
Communication	1	1	1	0	0	0	3	0.45
Community Care Coordination/Transitions of Care	1	0	0	0	0	0	1	0.15
Dental	0	0	1	1	0	0	2	0.30
Diabetes	29	3	17	0	2	0	51	7.59
Diagnostic Imaging	5	0	7	0	0	0	12	1.79
Ears, Nose, and Throat	6	1	0	0	1	3	11	1.64
Emergency Care	0	0	0	0	0	2	2	0.30
Eyes/Vision	3	0	7	0	0	0	10	1.49
Functional Status	13	0	0	0	0	0	13	1.93
Gastrointestinal	4	0	3	0	0	0	7	1.04
Health Services Administration	61	2	5	0	1	2	71	10.57
Health Status	2	0	11	0	0	0	13	1.93
Immunizations	31	1	2	0	32	0	66	9.82
Infant/Child Health	11	2	2	1	3	3	22	3.27
Mental Health Care & Substance-related Care	33	2	8	0	2	0	45	6.70
Mortality	18	0	0	0	0	0	18	2.68
Musculoskeletal	18	0	3	0	0	0	21	3.13
Nutrition & Exercise	4	1	0	0	2	0	7	1.04
Obesity	1	0	0	0	0	0	1	0.15
Pain	4	0	1	0	0	0	5	0.74
Patient Safety	79	0	6	0	3	3	91	13.54
Preventive Care	1	0	0	0	29	6	36	5.36
Public Health	3	0	1	1	0	0	5	0.74
Readmission	6	0	0	0	0	0	6	0.89
Renal & Genitourinary	16	0	3	0	0	0	19	2.83
Reproductive Health	12	1	2	0	2	0	17	2.53
Respiratory	22	0	5	0	1	0	28	4.17
Screening	27	2	8	0	3	2	42	6.25
Surgical Procedures	40	2	15	0	0	2	59	8.78
Women's Health	5	0	2	0	0	0	7	1.04

TOTAL	476	19	115	1	48	13	**672**	142.86
% of Grand Total	70.8	2.83	17.1	0.15	7.14	1.93	100	

The sum of the percentages listed in the rows exceeds 100% because 201 of the 672 measures observed (29.9%) were associated with more than one condition or topic.

1. National Quality Forum. Maximizing the Value of Measurement: MAP 2017 Guidance 2017.
2. Chassin MR, Loeb JM, Schmaltz SP, Wachter RM. Accountability measures--using measurement to promote quality improvement. N Engl J Med. 2010;363(7):683-8.
3. Forum NQ. Measure Evaluation Criteria. National Quality Forum [Internet]. 2011. Available from: http://www.qualityforum.org/docs/measure_evaluation_criteria.aspx.
4. Pritchard RD, Weaver SJ, Ashwood EL. Evidence-based productivity improvement: A practical guide to the productivity measurement and enhancement system. New York: Routledge Academic.; 2011.
5. Buchanan R. Wicked Problems in Design Thinking. 1992;8(2):5-21.
6. Wiggins GP, McTighe J. Understanding by design. Expanded 2nd ed. Alexandria, VA: Association for Supervision and Curriculum Development; 2005. xi, 370 p. p.
7. van der Geer E, van Tuijl HF, Rutte CG. Performance management in healthcare: performance indicator development, task uncertainty, and types of performance indicators. Soc Sci Med. 2009;69(10):1523-30.
8. DeNisi AS, Murphy KR. Performance appraisal and performance management: 100 years of progress? J Appl Psychol. 2017;102(3):421-33.
